# Tumor Microenvironment: Lactic Acid Promotes Tumor Development

**DOI:** 10.1155/2022/3119375

**Published:** 2022-06-12

**Authors:** Yuting Gao, Hao Zhou, Gege Liu, Junlu Wu, Yi Yuan, Anquan Shang

**Affiliations:** Department of Laboratory Medicine, Shanghai Tongji Hospital, School of Medicine, Tongji University, Shanghai 200065, China

## Abstract

Lactic acid is a “metabolic waste” product of glycolysis that is produced in the body. However, the role of lactic acid in the development of human malignancies has gained increasing interest lately as a multifunctional small molecule chemical. There is evidence that tumor cells may create a large amount of lactic acid through glycolysis even when they have abundant oxygen. Tumor tissues have a higher quantity of lactic acid than normal tissues. Lactic acid is required for tumor development. Lactate is an immunomodulatory chemical that affects both innate and adaptive immune cells' effector functions. In immune cells, the lactate signaling pathway may potentially serve as a link between metabolism and immunity. Lactate homeostasis is significantly disrupted in the TME. Lactate accumulation results in acidosis, angiogenesis, immunosuppression, and tumor cell proliferation and survival, all of which are deleterious to health. Thus, augmenting anticancer immune responses by lactate metabolism inhibition may modify lactate levels in the tumor microenvironment. This review will evaluate the role of lactic acid in tumor formation, metastasis, prognosis, treatment, and histone modification. Our findings will be of considerable interest to readers, particularly those engaged in the therapeutic treatment of cancer patients. Treatments targeting the inhibition of lactate synthesis and blocking the source of lactate have emerged as a potential new therapeutic option for oncology patients. Additionally, lactic acid levels in the plasma may serve as biomarkers for disease stage and may be beneficial for evaluating therapy effectiveness in individuals with tumors.

## 1. Introduction

When glycolysis breaks down lactic acid, it is regarded as a “metabolic waste.” However, it has since been shown that lactic acid in the heart, brain, and bones under physiological circumstances may be converted to glucose in the liver as an alternative energy source through the lactic acid cycle. Tumor cells use glucose as their primary energy source for fast multiplication and generate excess lactate through the glycolytic route even in the presence of adequate oxygen, according to findings from Warburg's research on lactate's involvement in tumor metabolism. Lactic acid in the tumor microenvironment (TME) improves tumor cell immune evasion by lowering immunological cell activity and the generation of inflammatory mediators. Tumor tissues have greater levels of lactate than normal tissues, and lactate aids in the development and spread of the tumor. Additionally, lactate may be used as a tumor prognostic biomarker as well as a treatment response biomarker and as a tumor therapeutic target. When it comes to tumor cell biology and prognosis, lactate is an important factor in tumor cell biology and prognosis. As a metabolite with a distinct signaling mechanism, it plays a role in tumor formation and can even affect epigenetics. Herein, the role of lactate in TME, tumor formation, metastasis, prognosis, and alterations in histone lactonization is discussed.

### 1.1. Lactic Acid Overview

Lactic acid is typically present in the human body as the L-lactic acid isomer, but it also occurs as the D-lactic acid isomer, which Carl discovered in 1780 [1, 2]. In normal tissues, the concentration of L-lactic acid ranges between 1.5 and 3 mM, while in tumor tissues, the concentration may range anywhere from 10 to 30 mM [3]. Tumor cells, on the other hand, get most of their energy mainly from the conversion of glucose to lactate via glycolysis, while most normal cells use glucose for oxidative phosphorylation to make ATP [4]. The majority of solid tumors generate lactate largely from glucose [5], while glutamine may also be converted enzymatically to lactate [6]. Many of the tricarboxylic acid (TCA) intermediates are produced from lactate when glucose is not available in the body, according to the findings of isotopic analysis using U-13C-labeled lactate [7]. Lactate is mostly produced by glycolysis-producing cancer cells and cancer-associated fibroblasts (CAF) in tumor tissue [8].

### 1.2. Lactic Acid and the Tumor Microenvironment (TME)

The TME is comprised of tumor cells, endothelial cells, CAF, and immune cells, as well as a noncancerous cellular matrix containing numerous peptide components (growth factors, chemokines, cytokines, and antibodies) [9]. Tumor cells must boost glucose uptake and glycolysis rate to meet the body's energy demands. Consequently, tumor cells may exert significant influence over energy consumption [10]. They do this by increasing the rate of glycolysis. Lactic acid affects a number of cellular activities inside the TME as an energy source, signaling molecule, and key tumor immunosuppressive factor [8]. A weak acidic environment in the extracellular pH of TME is caused by cancer cells' excessive use of glucose, resulting in a buildup of lactic acid, which in turn promotes tumor spreading, angiogenesis, treatment resistance, and immune suppression [11–13]. Meanwhile, lactic acid generation reduces the ability of NK and NKT cells to fight cancer, which promotes tumor growth [14, 15]. NKT cells are antitumor immune cells that produce cytokines that stimulate both natural and acquired immune responses [16]. The weak acidic environment in TME inhibits the release of inflammatory cytokines, which are required for T helper (TH) cell polarization and inflammatory dendritic cell (DC) differentiation [17]. According to the research of Fischer et al. [18], lactic acid increases immunosuppression and cancer growth by suppressing T lymphocyte proliferation and cytokines. Lin et al. [19] discovered that lactic acid inhibits DC cell differentiation and makes them tolerant. Meanwhile, Il-10 production rises, and Il-10 may evade NK cell immune monitoring. Tumor-associated macrophages (TAM) in TME come in two flavors: a traditional M1 phenotype that suppresses cancer cell proliferation and an M2 phenotype that performs the exact opposite function, promoting cancer cell growth and metastasis in the process. TAM function may be inhibited by tumor-derived lactate [20, 21], and lactate can increase breast cancer proliferation, migration, and angiogenesis by stimulating ERK/STAT3 signaling [17, 22], or M2 phenotypic polarization of macrophages was increased in a monocarboxylate transporter- (MCT-) dependent manner [23]. Lactate promotes the release of chemokine ligand 5 (CCL5) in a TME model of breast cancer cells, increasing cell motility and inducing cancer cell epithelial-to-mesenchymal transition. CAF can deliver lactate to oxidative tumor cells via TCA while also maintaining tumor growth and metastasis via mitochondrial oxidative phosphorylation, a phenomenon known as the anti-Warburg effect [24, 25]. CAF is a common cell type in tumor tissues and may play a role in tumor development, invasion, and metastasis via paracrine pathways [26]. CAF undergoes extensive glycolysis in the tumor microenvironment of lung cancer and promotes NSCLC cell epithelial-mesenchymal transition, migration, and invasion [27]. A possible way to impact the path of tumor growth is by altering the immunological status of tumor-infiltrating immune cells. The Warburg effect and the lactate shuttle lead to acidosis, angiogenesis, immunosuppression, and the proliferation and survival of tumor cells when lactate accumulates. Overall, lactic acid in TME promotes tumor growth by decreasing immune cell activity and allowing cancer cells to evade the body's natural defenses.

### 1.3. Lactic Acid and Tumor Growth, and Metastasis

Lactate is an endogenous ligand for the G protein-coupled receptor (GPR81), and their interaction may operate as a signaling agent through both autocrine and paracrine processes [28–30]. Lactate works as a signaling molecule by activating GPR81, which promotes tumor angiogenesis and immunological evasion. GPR81 has been discovered in colon, lung, salivary gland, hepatocyte, and breast cancer cell lines, as well as in breast cancer patients [31]. At sufficient concentrations, lactate may bind to GPR81 [32]. Lactate was found to promote tumor cell immune escape in breast cancer by activating the GPR81 receptor in TME, thereby inducing PD-L1 production and inhibiting tumor-specific antigen presentation by antigen-presenting cells to other immune cells [33], and a similar effect was found in lung cancer [34]. Furthermore, GPR81 may stimulate the production of endothelial cells in breast cancer cells, which influences angiogenesis [35]. Lactate also suppresses NF-*κ*B activation and the formation of inflammatory vesicles through GPR81 receptors on colon dendritic cells and macrophages, while increasing the production of proinflammatory cytokines IL-6, IL-1b, and TNF [36]. In recent research, lactate has been found to influence GPR81 expression in lung cancer through signal transduction and transcriptional activation (3snail3/STAT3) pathways [37]. Furthermore, GPR81 inhibitors (3-hydroxybutyrate, 3-OBA) and metformin may work together to limit cancer cell proliferation in vitro [38].

Most tumor-related fatalities are caused by metastases rather than the original tumor, which is one of the cancer's aggressive characteristics [39]. Tumor cells have been found to increase energy metabolism by regulating TCA and mitochondria-related pathways and responding quickly to metabolic cues in the environment, thus boosting tumor growth and metastasis [40]. According to Liu et al. [41], tumor cell-derived lactate activated the mammalian rapamycin complex 1, which inhibited the expression of TFEB and its downstream target genes, including Atp6v0d2, which encodes the vacuolar proton pump's D2 component (mTORC1). The Atp6v0d2 gene-deficient mice used in animal studies grew tumors more quickly than normal mice, and they also secreted more vascular endothelial growth factor (VEGF). In addition, postoperative survival was associated with high levels of Atp6v0d2 expression in lung cancer patients. Using melanoma (B16) and colon (MC38) cells, Toszka et al. [42] discovered that B16 cells had considerably greater glycolytic capacity, extracellular acidification, and H^+^ generation than MC38 cells; it was also shown in animal studies that melanoma had higher glycolytic activity than colon. Those results indicate that an acidic melanomic milieu may influence the expression of G protein-coupled receptor-dependent transcriptional repressors (ICERs) in TAM, encouraging TAM differentiation to an M2 noninflammatory phenotype, resulting in immune evasion and boosting tumor development. Aside from that, the macrophage-expressed lactate receptor GPR132 may control the contact between breast tumors and macrophages by encouraging the M2 type of macrophage conversion, which aids tumor dissemination [43]. High lactate concentrations are associated with higher lesion grades in human astrocytomas [44]. Lactate concentrations were shown to be substantially greater in metastatic areas than in nonmetastatic sites in cervical and colorectal malignancies [45, 46]. Head and neck tumors have been linked to increased tumor lactate concentrations in biopsies from individuals with head and neck tumors [3]. To summarize, lactic acid plays a role in tumor development and spread.

### 1.4. Lactic Acid in Tumor Treatment, Prognostic Monitoring

Patients' prognoses have been found to be substantially associated with lactate concentrations in cervical, lung, colorectal, breast, and head and neck tumor tissues [45, 47, 48].

#### 1.4.1. Tumor Prediction and Treatment Response Biomarkers Based on Lactate

A study by Park et al. [7] found that PI3K/mTOR pathway inhibitors reduced breast cancer cell proliferation in high glucose media, but these inhibitors had no effect when lactate served as the primary metabolic substrate. This suggests that cancer cells can use lactate to reduce their reliance on glycolysis for energy. NSCLC patients who use tyrosine kinase inhibitors for an extended period may have an increased generation of lactate by their tumor cells, which stimulates TME cells to produce more hepatocyte growth factor (HGF), further increasing cancer resistance and development. Another study found that lactic acidification-induced cAMP-dependent signaling in tumors might be regarded as an effective immunosuppressive mechanism, providing fresh techniques and therapeutic targets for tumor immunotherapy [42]. This indicates that monitoring variations in lactate levels may provide some relevant clinical information for tumor prognosis and therapy choices [49]. To summarize, tumor lactate levels may be used to predict prognosis and therapy success.

#### 1.4.2. Lactic Acid as a Monitoring Marker for Tumor Treatment

Lactate is excreted by glycolytic cells in tumors and reabsorbed by oxidative cancer cells to fuel the tricarboxylic acid (TCA) cycle after conversion to pyruvate [50]. MCTs mediate these proton-linked lactate fluxes and are thus ideal candidates for interfering with the lactate shuttle and inhibiting tumor development [50]. MCT1 expression is increased in tumors and is associated with a poor prognosis in cancer patients, and inhibiting lactate transport with AR-C155858, an MCT1 inhibitor, restores immune cells' capacity to generate IFN-*γ* [51, 52]. Additionally, AR-C155858 dramatically decreased tumor development in breast cancer-bearing animals [53], and metformin (metformin) blocked MCT1 activity, hence preventing intracellular lactate buildup [54]. According to one study, anti-CTLA-4 antibody therapy improved the prognosis of mice with glycolysis-deficient tumors, indicating that decreased tumor competition for glucose may enhance the therapeutic effect of CTLA-4 blockers [40]. Lactate secreted by tumor cells increased the transcription of IL-23p19 and IL-23 in monocytes/macrophages and tumor-infiltrating immune cells, implying that lactate is not only a terminal metabolite but also a proinflammatory mediator, and thus, targeting the lactate-induced proinflammatory response may be a therapeutic strategy for cancer [55]. 7ACC significantly inhibited both lactate inward flow and cell proliferation in human cervical cancer cells SiHa and Hela expressing both MCT1 and MCT4 isoforms, but the genuine MCT1/MCT2 inhibitor AR-C155858 had no impact [50]. Lactate significantly enhances tumor cell motility in a dose-dependent manner in head and neck cancer cell lines, and high lactate concentrations facilitate tumor immune escape and promote migration of malignant cell populations, thereby promoting tumor progression. Lactate can also be used as an indicator of tumor metastasis and a poor overall survival prognosis in patients [56]. Cancer immunotherapy with oral bicarbonate or bicarbonate in combination with anti-CTLA-4 and anti-PD-1 enhances the antitumor immune response [57, 58]. Checkpoint-blocking antibodies targeting CTLA-4, PD-1, and PD-L1 may, on the other hand, promote T cell glycolysis and IFN production by restoring pH in the TME [59]. Consequently, LDH-A increases antitumor immunity in bone marrow cells by stimulating Th17 cells to control angiogenesis and PDL1 expression; consequently, LDH-A inhibitors may provide a unique strategy for increasing the effectiveness of checkpoint inhibitors [60]. Dichloroacetate (DCA) decreases tumor cell glycolysis in melanoma [61] and also enhances the immunosuppressive state generated by lactate, hence increasing the efficacy of anticancer immunotherapy.

Pyruvate to lactate interconversion is regulated by lactate dehydrogenase (LDH), which is a NAD^+^ oxidoreductase. Tetrameric enzymes such as LDH may join M and H protein subunits in human tissues to create five different kinds of tetramers, such as LDH-1 (4H), LDH-2 (3H1M), LDH-3 (2H2M), LDH-4 (1H3M), and LDH-5 (4M). Tetramers of these enzymes can also be found in other organisms, such as bacteria and fungi [62]. While LDH1 is the predominant form of LDH in cardiac muscle, where it reduces lactate to pyruvate and oxidizes NAD+ thermodynamically to NAD+, LDH5 is the predominant form of LDH in skeletal muscle [63]. As demonstrated, lactate synthesis may be stopped, and tumor development is inhibited by inhibiting LDH1 enzyme activity; LDH1 inhibitors are needed for the treatment of cancer, and they must be found and developed quickly. According to Zhou et al. [64], compound 24C not only suppressed LDH1 activity and decreased lactate generation but also switched the metabolic route from glycolysis to oxidative phosphorylation, increasing the oxygen consumption rate of cancer cells. An efficient LDH1 inhibitor promoted metabolic reprogramming in MG-63 cancer cells from glycolysis to mitochondrial aerobic respiration, increasing cell death and suppressing growth, decreasing cell survival. As a result, chemicals 24C and 11C may suppress tumor growth and have potential anticancer therapeutic applications [18]. Selenobenzene analog PSTMB decreased LDH1 activity and reduced lactate generation in a number of tumor cells, including lung cancer cells (NCI-H460), breast cancer cells (MCF-7), hepatocellular carcinoma cells (Hep3B), malignant melanoma cells (A375), colorectal cancer cells (HT-29), and mouse lung cancer cells (LLC) [65]. Besides gossypol (AT-101), the LDH1 isomer is also inhibited by FX-11 and N-hydroxyindole analogs of cottonellol (AT-101) [66]. As a result, anticancer applications for selenobenzenes, cottonellol, and their derivatives seem quite promising. Tumors may be effectively treated by preventing the production of lactate and preventing its absorption. The anticancer compound 7ACC2 was found to be a potent MPC inhibitor that not only blocked the uptake of extracellular lactate continuously by promoting the accumulation of pyruvate in the cell and preventing glucose oxidative metabolism but also improved the sensitivity of transplanted tumors to radiation therapy [67]. The significance of lactate in tumorigenesis, progression, and treatment resistance suggests that inhibiting lactate production and blocking lactate sources may be a viable approach for treating cancer.

## 2. Lactic Acidification Modifications and their Functions

Several epigenetic mechanisms, such as histone posttranslational changes (such as acetylation, methylation, and phosphatidylinositol-phosphate phosphorylation), are critical in maintaining the delicate balance between the active and inactive states of chromatin. Histone posttranslational changes have been linked to tumor formation and progression in many studies, including lung, colorectal, breast, and prostate malignancies [68–70]. Lactylation modification of histones, a novel histone posttranslational modification discovered recently, regulates gene transcription [71]. In order to mimic a Gram-negative bacterial infection, Zhang et al. [71] stimulated mouse bone marrow-derived macrophages with lipopolysaccharide and interferon (LPS+IFN-*γ*) under hypoxic conditions. The macrophages responded quickly to the inflammatory infection, producing large amounts of lactate and inducing lactylation modification of histones within 16-24 hours of stimulation, while histone acetylation decreased. Lactylation was shown to be labeled to histones later than acetylation using isotope labeling of glucose metabolism, indicating that the two modifications have distinct purposes. To find out whether histone lactylation was linked to the expression of genes involved in cellular homeostasis (such as wound healing pathways), the researchers used the ChIP-seq and RNA-seq approaches. According to the findings presented here, lactonization was shown to modify a histone lysine site for the first time. Macrophage-specific production of B-cell adapter for PI3K (BCAP) inhibits the activity of important downstream proteins GSK3b and FOXO1, according to other research employing a BCAP-deficient animal model, which leads to reduced inflammation in the organism [72]. This research also discovered that BCAP-deficient macrophages had lower lactate concentration, which reduces histone lactonization changes, which in turn affects negatively the expression of damage repair genes (Arg1 and Klf4), reducing the transformation of repair macrophages. Taken together, these data show that histone lactylation changes play a crucial role in the formation of tumors and the control of the immune system.

## 3. Conclusion and Future Prospects

**Figure 1:**
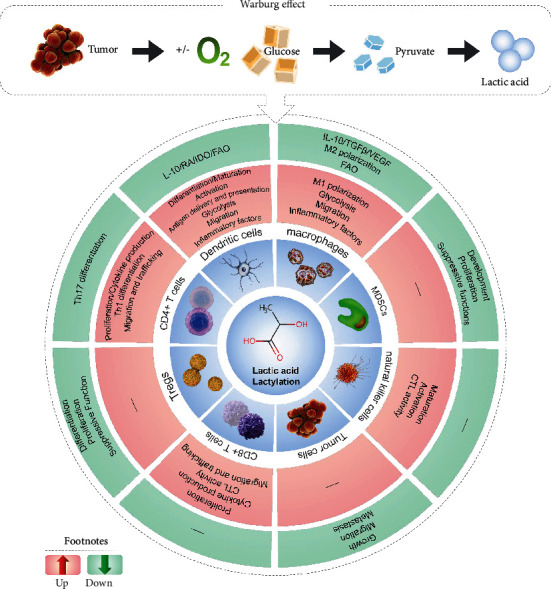
Lactic acid/lactylation regulates tumor-associated immune cell mechanisms.

As shown in this review, studies to understand the mechanisms of lactate play an important role in oncology diagnosis and treatment. Histone lactylation regulates gene expression via epigenetic means. To be sure, it will be fascinating to figure out how lactate metabolism affects epigenetic programming under various cellular conditions/environment. For example, the mechanisms by which the tumor microenvironment causes metabolite changes, the effects of metabolite changes on epigenetic modifying complexes and epigenetic events, and the mechanisms by which metabolite-sensitive epigenetic events are translated into specific cell differentiation gene programs are among the mechanistic series of events that need to be better defined (Figure 1).

Lactic acid is involved in TME, tumor growth and metastasis, prognosis, therapy, and histone modification. High amounts of lactic acid in tumor tissue are connected to tumor growth, metastasis, and long-term prognosis. Acidification of TME may have a profound influence on tumor malignancy and progression. Lactate's interaction with tumor-infiltrating immune cells is unknown, but researchers believe it might be a starting point for future cancer therapies. Compound 7ACC2, which slows extracellular lactate absorption, has hazardous side effects that have not been totally eliminated. Tumor treatment may include inhibiting lactate generation. New anticancer drugs have minimal toxicity and are simple to synthesis, but in vivo validation is needed to determine their full biological potential. Histone lactonization modifications have also supplied new insights into lactate and the “Warburg” effect, a tumor research process. As research and detection tools improve, lactate's role in tumor development will become clearer. Future investigations on lactate immunometabolism may provide innovative drugs to modulate immune cell activity more selectively and with fewer side effects. There will also be breakthroughs in tumor identification and treatment targeted concepts and techniques.

## References

[1] Talasniemi J. P., Pennanen S., Savolainen H., Niskanen L., Liesivuori J. (2008). Analytical investigation: assay of D-lactate in diabetic plasma and urine. *Clinical Biochemistry*.

[2] Watson N. C., Heard S. O. (2010). The use of lactate as a biomarker. *Journal of Intensive Care Medicine*.

[3] Brizel D. M., Schroeder T., Scher R. L. (2001). Elevated tumor lactate concentrations predict for an increased risk of metastases in head-and-neck cancer. *International Journal of Radiation Oncology • Biology • Physics*.

[4] van der Bliek A. M., Sedensky M. M., Morgan P. G. (2017). Cell biology of the mitochondrion. *Genetics*.

[5] Liberti M. V., Locasale J. W. (2016). The Warburg effect: how does it benefit cancer cells?. *Trends in Biochemical Sciences*.

[6] DeBerardinis R. J., Mancuso A., Daikhin E. (2007). Beyond aerobic glycolysis: transformed cells can engage in glutamine metabolism that exceeds the requirement for protein and nucleotide synthesis. *Proceedings of the National Academy of Sciences of the United States of America*.

[7] Park S., Chang C. Y., Safi R. (2016). ERR*α*-regulated lactate metabolism contributes to resistance to targeted therapies in breast cancer. *Cell Reports*.

[8] Ippolito L., Morandi A., Giannoni E., Chiarugi P. (2019). Lactate: a metabolic driver in the tumour landscape. *Trends in Biochemical Sciences*.

[9] Lyssiotis C. A., Kimmelman A. C. (2017). Metabolic interactions in the tumor microenvironment. *Trends in Cell Biology*.

[10] Hanahan D., Weinberg R. A. (2011). Hallmarks of cancer: the next generation. *Cell*.

[11] Xiong R., Xu R. X., Huang C., De Smedt S., Braeckmans K. (2021). Stimuli-responsive nanobubbles for biomedical applications. *Chemical Society Reviews*.

[12] Nguyen V. N., Yan Y., Zhao J., Yoon J. (2021). Heavy-atom-free photosensitizers: from molecular design to applications in the photodynamic therapy of cancer. *Accounts of Chemical Research*.

[13] Huang H., Wang X., Wang W. (2022). Injectable hydrogel for postoperative synergistic photothermal-chemodynamic tumor and anti-infection therapy. *Biomaterials*.

[14] Kumar A., Pyaram K., Yarosz E. L. (2019). Enhanced oxidative phosphorylation in NKT cells is essential for their survival and function. *Proceedings of the National Academy of Sciences of the United States of America*.

[15] Xie D., Zhu S., Bai L. (2016). Lactic acid in tumor microenvironments causes dysfunction of NKT cells by interfering with mTOR signaling. *Science China. Life Sciences*.

[16] Bae E. A., Seo H., Kim I. K., Jeon I., Kang C. Y. (2019). Roles of NKT cells in cancer immunotherapy. *Archives of Pharmacal Research*.

[17] Nasi A., Fekete T., Krishnamurthy A. (2013). Dendritic cell reprogramming by endogenously produced lactic acid. *The Journal of Immunology*.

[18] Fischer K., Hoffmann P., Voelkl S. (2007). Inhibitory effect of tumor cell-derived lactic acid on human T cells. *Blood*.

[19] Lin S., Sun L., Lyu X. (2017). Lactate-activated macrophages induced aerobic glycolysis and epithelial-mesenchymal transition in breast cancer by regulation of CCL5-CCR5 axis: a positive metabolic feedback loop. *Oncotarget*.

[20] Colegio O. R., Chu N. Q., Szabo A. L. (2014). Functional polarization of tumour-associated macrophages by tumour-derived lactic acid. *Nature*.

[21] Selleri S., Bifsha P., Civini S. (2016). Human mesenchymal stromal cell-secreted lactate induces M2-macrophage differentiation by metabolic reprogramming. *Oncotarget*.

[22] Mu X., Shi W., Xu Y. (2018). Tumor-derived lactate induces M2 macrophage polarization via the activation of the ERK/STAT3 signaling pathway in breast cancer. *Cell Cycle*.

[23] Zhang J., Muri J., Fitzgerald G. (2020). Endothelial lactate controls muscle regeneration from ischemia by inducing M2-like macrophage polarization. *Cell Metabolism*.

[24] Bonuccelli G., Tsirigos A., Whitaker-Menezes D. (2010). Ketones and lactate "fuel" tumor growth and metastasis: evidence that epithelial cancer cells use oxidative mitochondrial metabolism. *Cell Cycle*.

[25] Pavlova N. N., Thompson C. B. (2016). The emerging hallmarks of cancer metabolism. *Cell Metabolism*.

[26] Shan T., Chen S., Chen X. (2017). Cancer-associated fibroblasts enhance pancreatic cancer cell invasion by remodeling the metabolic conversion mechanism. *Oncology Reports*.

[27] Luo M., Luo Y., Mao N. (2018). Cancer-associated fibroblasts accelerate malignant progression of non-small cell lung cancer via connexin 43-formed unidirectional gap junctional intercellular communication. *Cellular Physiology and Biochemistry : International Journal of Experimental Cellular Physiology, Biochemistry, and Pharmacology*.

[28] Brown T. P., Ganapathy V. (2020). Lactate/GPR81 signaling and proton motive force in cancer: role in angiogenesis, immune escape, nutrition, and Warburg phenomenon. *Pharmacology & Therapeutics*.

[29] Cai T. Q., Ren N., Jin L. (2008). Role of GPR81 in lactate-mediated reduction of adipose lipolysis. *Biochemical and Biophysical Research Communications*.

[30] Liu C., Wu J., Zhu J. (2009). Lactate inhibits lipolysis in fat cells through activation of an orphan G-protein-coupled receptor, GPR81. *The Journal of biological chemistry*.

[31] Stäubert C., Broom O. J., Nordström A. (2015). Hydroxycarboxylic acid receptors are essential for breast cancer cells to control their lipid/fatty acid metabolism. *Oncotarget*.

[32] Fliesser M., Morton C. O., Bonin M. (2015). Hypoxia-inducible factor 1*α* modulates metabolic activity and cytokine release in anti-Aspergillus fumigatus immune responses initiated by human dendritic cells. *International journal of medical microbiology: IJMM*.

[33] Brown T. P., Bhattacharjee P., Ramachandran S. (2020). The lactate receptor GPR81 promotes breast cancer growth via a paracrine mechanism involving antigen-presenting cells in the tumor microenvironment. *Oncogene*.

[34] Feng J., Yang H., Zhang Y. (2017). Tumor cell-derived lactate induces TAZ-dependent upregulation of PD-L1 through GPR81 in human lung cancer cells. *Oncogene*.

[35] Lee Y. J., Shin K. J., Park S. A. (2016). G-protein-coupled receptor 81 promotes a malignant phenotype in breast cancer through angiogenic factor secretion. *Oncotarget*.

[36] Ranganathan P., Shanmugam A., Swafford D. (2018). GPR81, a cell-surface receptor for lactate, regulates intestinal homeostasis and protects mice from experimental colitis. *The Journal of immunology: official journal of the American Association of Immunologists*.

[37] Xie Q., Zhu Z., He Y. (2020). A lactate-induced Snail/STAT3 pathway drives GPR81 expression in lung cancer cells. *Molecular basis of disease*.

[38] Chen S., Zhou X., Yang X. (2021). Dual blockade of lactate/GPR81 and PD-1/PD-L1 pathways enhances the anti-tumor effects of metformin. *Biomolecules*.

[39] Watson M. J., Vignali P., Mullett S. J. (2021). Metabolic support of tumour-infiltrating regulatory T cells by lactic acid. *Nature*.

[40] Zappasodi R., Serganova I., Cohen I. J. (2021). CTLA-4 blockade drives loss of T (reg) stability in glycolysis-low tumours. *Nature*.

[41] Lambert A. W., Pattabiraman D. R., Weinberg R. A. (2017). Emerging biological principles of metastasis. *Cell*.

[42] Morandi A., Giannoni E., Chiarugi P. (2016). Nutrient exploitation within the tumor-stroma metabolic crosstalk. *Trends in cancer*.

[43] Liu N., Luo J., Kuang D. (2019). Lactate inhibits ATP6V0d2 expression in tumor-associated macrophages to promote HIF-2*α*-mediated tumor progression. *The Journal of Clinical Investigation*.

[44] Bohn T., Rapp S., Luther N. (2018). Tumor immunoevasion via acidosis-dependent induction of regulatory tumor-associated macrophages. *Nature immunology*.

[45] Chen P., Zuo H., Xiong H. (2017). Gpr132 sensing of lactate mediates tumor-macrophage interplay to promote breast cancer metastasis. *Proceedings of the National Academy of Sciences of the United States of America*.

[46] Nikoobakht M., Shamshiripour P., Azimi Nekoo Z., Fallah Haghmohammadi S. (2019). Elevated lactate and total protein levels in stereotactic brain biopsy specimen; potential biomarkers of malignancy and poor prognosis. *Archives of Iranian Medicine*.

[47] Walenta S., Chau T. V., Schroeder T. (2003). Metabolic classification of human rectal adenocarcinomas: a novel guideline for clinical oncologists. *Journal of Cancer Research and Clinical Oncology*.

[48] Walenta S., Wetterling M., Lehrke M. (2000). High lactate levels predict likelihood of metastases, tumor recurrence, and restricted patient survival in human cervical cancers. *Cancer Research*.

[49] Vlachostergios P. J., Oikonomou K. G., Gibilaro E., Apergis G. (2015). Elevated lactic acid is a negative prognostic factor in metastatic lung cancer. *Cancer biomarkers: section A of Disease markers*.

[50] Draoui N., Schicke O., Seront E. (2014). Antitumor activity of 7-aminocarboxycoumarin derivatives, a new class of potent inhibitors of lactate influx but not efflux. *Molecular Cancer Therapeutics*.

[51] Payen V. L., Mina E., Van Hée V. F., Porporato P. E., Sonveaux P. (2020). Monocarboxylate transporters in cancer. *Molecular metabolism*.

[52] Sun X., Wang M., Wang M. (2020). Role of proton-coupled monocarboxylate transporters in cancer: from metabolic crosstalk to therapeutic potential. *Frontiers in cell and developmental biology*.

[53] Raychaudhuri D., Bhattacharya R., Sinha B. P. (2019). Lactate induces pro-tumor reprogramming in intratumoral plasmacytoid dendritic cells. *Frontiers in Immunology*.

[54] Sun H., Zhu A., Zhou X., Wang F. (2017). Suppression of pyruvate dehydrogenase kinase-2 re-sensitizes paclitaxel-resistant human lung cancer cells to paclitaxel. *Oncotarget*.

[55] Shime H., Yabu M., Akazawa T. (2008). Tumor-secreted lactic acid promotes IL-23/IL-17 proinflammatory pathway. *The Journal of immunology: official journal of the American Association of Immunologists*.

[56] Goetze K., Walenta S., Ksiazkiewicz M., Kunz-Schughart L. A., Mueller-Klieser W. (2011). Lactate enhances motility of tumor cells and inhibits monocyte migration and cytokine release. *International Journal of Oncology*.

[57] Pilon-Thomas S., Kodumudi K. N., El-Kenawi A. E. (2016). Neutralization of tumor acidity improves antitumor responses to immunotherapy. *Cancer Research*.

[58] Pötzl J., Roser D., Bankel L. (2017). Reversal of tumor acidosis by systemic buffering reactivates NK cells to express IFN-*γ* and induces NK cell-dependent lymphoma control without other immunotherapies. *International Journal of Cancer*.

[59] Chang C. H., Qiu J., O’Sullivan D. (2015). Metabolic competition in the tumor microenvironment is a driver of cancer progression. *Cell*.

[60] Kumar R., Mendonca J., Owoyemi O. (2021). Supraphysiologic testosterone induces ferroptosis and activates immune pathways through nucleophagy in prostate cancer. *Cancer Research*.

[61] Ohashi T., Akazawa T., Aoki M. (2013). Dichloroacetate improves immune dysfunction caused by tumor-secreted lactic acid and increases antitumor immunoreactivity. *International Journal of Cancer*.

[62] Walenta S., Schroeder T., Mueller-Klieser W. (2004). Lactate in solid malignant tumors: potential basis of a metabolic classification in clinical oncology. *Current Medicinal Chemistry*.

[63] Apicella M., Giannoni E., Fiore S. (2018). Increased lactate secretion by cancer cells sustains non-cell-autonomous adaptive resistance to MET and EGFR targeted therapies. *Cell Metabolism*.

[64] Adeva-Andany M., López-Ojén M., Funcasta-Calderón R. (2014). Comprehensive review on lactate metabolism in human health. *Mitochondrion*.

[65] Liang X., Liu L., Fu T. (2016). Exercise inducible lactate dehydrogenase B regulates mitochondrial function in skeletal muscle. *The Journal of biological chemistry*.

[66] Le A., Cooper C. R., Gouw A. M. (2010). Inhibition of lactate dehydrogenase A induces oxidative stress and inhibits tumor progression. *Proceedings of the National Academy of Sciences of the United States of America*.

[67] Zhou Y., Tao P., Wang M. (2019). Development of novel human lactate dehydrogenase A inhibitors: high-throughput screening, synthesis, and biological evaluations. *European Journal of Medicinal Chemistry*.

[68] Audia J. E., Campbell R. M. (2016). Histone modifications and cancer. *Cold Spring Harbor Perspectives in Biology*.

[69] Corbet C., Bastien E., Draoui N. (2018). Interruption of lactate uptake by inhibiting mitochondrial pyruvate transport unravels direct antitumor and radiosensitizing effects. *Nature Communications*.

[70] Kim E. Y., Chung T. W., Han C. W. (2019). A novel lactate dehydrogenase inhibitor, 1-(phenylseleno)-4-(trifluoromethyl) benzene, suppresses tumor growth through apoptotic cell death. *Scientific Reports*.

[71] Zhang D., Tang Z., Huang H. (2019). Metabolic regulation of gene expression by histone lactylation. *Nature*.

[72] Abbaoui B., Telu K. H., Lucas C. R. (2017). The impact of cruciferous vegetable isothiocyanates on histone acetylation and histone phosphorylation in bladder cancer. *Journal of Proteomics*.

